# Mediating effect of sequential memory on the relationship between visual-motor integration and self-care performance in young children with autism spectrum disorder

**DOI:** 10.3389/fpsyg.2022.988493

**Published:** 2022-10-04

**Authors:** Ling-Yi Lin, I-Jou Chi, Yi-Shan Sung

**Affiliations:** ^1^Department of Occupational Therapy, College of Medicine, National Cheng Kung University, Tainan, Taiwan; ^2^Institute of Allied Health Sciences, College of Medicine, National Cheng Kung University, Tainan, Taiwan; ^3^Institute of Brain Science, School of Medicine, National Yang Ming Chiao Tung University, Taipei, Taiwan

**Keywords:** autism spectrum disorder, motor-free visual perception, sequential memory, visual-motor integration, self-care

## Abstract

**Objective:**

Visual perception is a skill that contributes to the performance of self-care and important development tasks in early childhood. The relationship between self-care and visual perception is especially significant for young children with autism spectrum disorder (ASD), who have been described as visual learners. However, this relationship is not clearly understood among young children with ASD. We investigated the role of motor-free visual perception on the relationship between self-care and visual-motor integration in young children with ASD.

**Methods:**

A sample of 66 children with ASD aged 48 to 83 months were recruited. Measurements included the Assessment of Motor and Process Skills, the Developmental Test of Visual Perception—Third Edition, and Test of Visual-Perceptual Skills—Third Edition.

**Results:**

The results indicated that self-care performance had significant positive correlations with visual-motor integration, visual discrimination, visual memory, visual spatial relationships, and visual sequential memory. Of these, visual sequential memory and visual spatial relationships were the main factors related to self-care performance. Sequential memory was a mediator of the relationship between visual-motor integration and self-care performance.

**Conclusion:**

This study establishes a deeper understanding of self-care and motor-free visual perception among young children with ASD. Understanding the relationship between visual perception and self-care in young children with ASD may aid professionals in providing self-care interventions for this population.

## Introduction

Autism spectrum disorder (ASD) is a lifelong developmental condition that can present challenges with a person’s performance skills (American Psychiatric Association, 2013). Research suggests that individuals with ASD experience problems related to sensory processing and motor skills, including issues with sensory integration, sensory over-responsivity (Green et al., 2011; Sanz-Cervera et al., 2019), balance problems, clumsy motor patterns, and poor performance in both gross and fine motor skills (Isabel, 2004; Lloyd et al., 2011; Stins and Emck, 2018; WHO, 2022). Difficulties in performance in these skills may relate to difficulties performing jobs in later life. Most cases of ASD are diagnosed at preschool age and have a lifelong impact, thus highlighting the need to explore the performance skills of individuals with ASD at a young age.

Visual perception is a basic ability that requires the individual to receive, interpret, and act upon specific visual stimuli. Generally, visual perceptual skills include the following eight subcategories: visual attention, visual discrimination, visual spatial relationships, visual memory, visual sequential memory, form constancy, foreground-background differentiation (figure-ground), and visual closure (Martin, 2006). Visual-motor integration primarily refers to copying ability and to hand-eye coordination (Hammill et al., 2014). Sound visual-motor integration should rely on good visual perception skills (Martin, 2006; Hammill et al., 2014). Critically, visual perception abilities contribute to the performance of occupational tasks, as well as exploring and interacting with the world (Kurtz, 2006; Martin, 2006). Furthermore, visual perception problems may also be reflected in social interaction difficulties (Webb et al., 2017).

Individuals with ASD demonstrate different visual perception patterns from those without ASD (Dakin and Frith, 2005; Kurtz, 2006; Keehn and Joseph, 2008; Sabatino DiCriscio and Troiani, 2018). Although visual acuity in children with ASD is generally considered in the expected range (Anketell et al., 2015), special visual preferences and characteristics of individuals with ASD differ from those without ASD (Dakin and Frith, 2005). Individuals with ASD usually focus on fine details, also known as local structure; have weak processing ability for global structure (e.g., the broader picture); and find it hard to ignore irrelevant visual information in their surroundings (Dakin and Frith, 2005). Previous studies have proposed some atypical characteristics of visual processing patterns in individuals with ASD (Simmons et al., 2009; Bakroon and Lakshminarayanan, 2016). Infants who are later diagnosed with ASD have similar visual perception patterns as other infants (Hyvarinen et al., 2014). However, the visual perception patterns of school-age children, adolescents, and adults with ASD are different from those in individuals without ASD (Dakin and Frith, 2005; Kurtz, 2006; Keehn and Joseph, 2008; Wegiel et al., 2015). To date, only a few neurological studies have investigated the visual perception of young children with ASD due to the limitations of the methodologies (Schumann et al., 2010; Wegiel et al., 2015). Little is known about specific visual perception performance in children with ASD aged between 4 and 6. Further research is needed to determine whether visual perception is a problem that affects performance skills in young children with ASD.

Interventions and training programs for self-care skills for ASD mainly use visually assisted learning strategies (Carothers and Taylor, 2004; Jasmin et al., 2008; Suprajitno and Arisky, 2019), such as picture schedules, video modeling, demonstration, imitation, and observational learning (Carothers and Taylor, 2004; Kenworthy et al., 2008; Vetrayan et al., 2015; Aldi et al., 2016; Brown et al., 2019). These strategies for learning and performing self-care skills greatly rely on visual perception, including aspects of visual discrimination, visual memory, visual sequential memory, figure-ground, visual closure, and eye-hand coordination (Martin, 2006; Hammill et al., 2014; James et al., 2015). People with difficulties in visual perceptual skills experience difficulties in performing self-care tasks (Parush et al., 1998). However, in these previous studies, researchers used motor-free evaluations to assess visual perception (Elbasan et al., 2011; James et al., 2015). While Jasmin et al. (2008) showed that a significant correlation existed between visual-motor integration and self-care in preschool children with ASD, their research did not include motor-free visual perception. To obtain a comprehensive result, Chi and Lin (2020) demonstrated that significant relationships exist between self-care performance, motor-free visual perception, and visual-motor integration abilities in young children with ASD. However, the relationship between specific motor-free visual perception abilities and self-care performance has not been confirmed in young children with ASD, and so the role of motor-free visual perception ability in the performance of self-care tasks remains unclear. Further examination of these relationships in young children with ASD is therefore needed.

The present study was a secondary data analysis of Chi and Lin (2020) and looking at whether specific motor-free visual perception abilities were associated with visual-motor integration ability and self-care performance in young children with autism. In this study, we address the following questions: (a) Are the specific motor-free visual perception abilities of young children with ASD correlated with visual-motor integration ability and self-care performance? (b) To what extent does the motor-free visual perception ability predict the self-care performance of young children with ASD? (c) Is motor-free visual perception a potential mediator of the relationship between visual-motor integration ability and self-care performance?

## Materials and methods

### Participants

This study was a secondary analysis of data from the research carried out by Chi and Lin (Chi and Lin, 2020). A sample of 66 young children with ASD (aged 48–83 months old) were recruited from a medical center in Tainan, Taiwan. The majority of the sample were boys (86.4%), with a mean age of 62.9 months. Most children were aged 60–71 months (60.6%), followed by 48–59 months (30.3%), and 73–83 years (9.1%). The children had been diagnosed with ASD by registered pediatric psychiatrists according to DSM-5 criteria (American Psychiatric Association, 2013) and had an average score of 33.5 (SD = 2.2) on the Standard Version of Childhood Autism Rating Scale—Second Edition (Schopler and Al, 2010). Their cognitive ability ranged from mild to superior levels [intelligence quotient (IQ) of 67 to 127] on the Wechsler Preschool and Primary Scale of Intelligence, with the mean score of the IQ at 85.6 (SD = 18.5). About 14.8% of the participants had an IQ score lower than 70. The sample characteristics, self-care, and partial visual perception data from these children with ASD have been reported previously (Chi and Lin, 2020).

### Instruments

#### Assessment of motor and process skills (AMPS)

The AMPS is an objective, standardized, observation-based self-care measurement (Fisher and Jones, 2012). The AMPS includes normative data for various population groups and is recommended for use with any person over the age of two years with any functional limitation. Activities of daily living (ADL), including self-feeding, dressing, personal hygiene, grooming, and bathing, were used to assess the quality of self-care performance in young children by a trained and certified rater. The scoring consists of ADL motor skills and ADL process skills. The score for each unit ranges from 1 (severely deficit performance) to 4 (competent performance). By using the AMPS computer software, the motor and process skills scores for each child were calculated. The AMPS log-odds probability unit (logit) scores can be compared to the normative range of self-care abilities typically seen among healthy individuals of the same age (Fisher and Jones, 2012). Cut-off scores of 1.5 logits on motor skills and 1 logit on process skills were indicated for determining the need for assistance (Merritt, 2011). Higher logits represent more competent self-care performance. The AMPS shows good reliability and validity (Fisher, 1993; Merritt, 2011).

#### Developmental test of visual perception—Third edition (DTVP-3)

The DTVP-3 is a standardized measure to evaluate the motor-reduced visual perception, visual-motor integration, and general visual perception performance of children between the ages of 4 and 12 years (Hammill et al., 2014). This study used the visual-motor integration (VMI) section of the DTVP-3, which consists of copying and eye-hand coordination tasks. Scaled scores, percentile rank, and age equivalent scores can be generated by age norms. The VMI composite scores, age-based standardized scores with a mean of 100, were used for data analysis. Higher scores indicate better VMI performance. The DTVP-3 has good reliability and criterion validity (Hammill et al., 2014). Based on the DTVP-3 test manual, the mean VMI composite score for children with ASD is 90 (Hammill et al., 2014).

#### Test of visual perceptual skills—Third edition (TVPS-3)

The TVPS-3 is a standardized motor-free visual perceptual skills in children and young people aged 4 to 18 years (Martin, 2006). The TVPS-3 includes seven motor-free visual perceptual subtests: visual discrimination, visual spatial relationships, visual memory, visual sequential memory, form constancy, figure-ground, and visual closure. Each subtest score ranges from 0 to 16. Higher scores indicate better performance of visual perceptual skills. The scaled scores of each subtest according to age norms were used for data analysis. Good internal consistency, great test–retest reliability, and good criterion-related validity were reported previously (Martin, 2006).

### Procedures

This study was approved by the Institutional Review Board of National Cheng Kung University Hospital. The details of the procedures have been described previously (Chi and Lin, 2020).

### Data analysis

This study used the logits of the AMPS, the VMI composite scores of the DTVP-3, and the subtest scaled scores of the TVPS-3. SPSS 22.0 was used to analyze descriptive statistics for study variables. Pearson correlation matrices were used to examine the relationship between self-care, visual-motor integration, and the visual perception subtests. The motor-free visual perception subtests were selected for inclusion in stepwise multiple regression analysis to determine the extent of the relationship among variables. A F-to-Enter significance level to be less than the 0.05 level and a F-to-Remove significance level to be greater than the 0.10 level were conducted. The method outlined by Hayes (2012) was followed to examine whether motor-free visual perception was a mediating variable that could account for the relationship between self-care and visual-motor integration. Analyses using Sobel’s test (Preacher and Hayes, 2004) were used to test the mediation effect. The level of significance was set at *p* < 0.05.

## Results

Table 1 presents the mean scores and correlation coefficients of the self-care and visual perception subtests. Children with ASD had a mean score of 1.34 logits on the AMPS ADL motor skills and 0.56 logits on the AMPS ADL process skills. The mean VMI composite score was 93.98. The scores ranged from below average to average among all motor-free visual perception subtests. Significant low to moderate positive correlations were found between self-care performance, visual-motor integration, and some motor-free visual perception subtests. Children with ASD who had greater visual-motor integration, visual discrimination, visual memory, visual spatial relationships, and visual sequential memory demonstrated better self-care performance. No correlations were evident between self-care performance and the form constancy, figure-ground, or visual closure subtests. Partial correlation analysis was employed to detect possible effects of IQ and age on the relationship between self-care performance, visual-motor integration, and motor-free visual perception. The low to moderate positive correlations between the AMPS ADL motor skills, AMPS ADL process skills, visual-motor integration, visual spatial relationships, and visual sequential memory remained significant after controlling the effects of IQ and age.

**Table 1:** Mean scores and coefficients of self-care and visual perception subtests (*n* = 66).

Subtests	Mean ± SD	1	2	3	4	5	6	7	8	9	10
1. AMPS: motor skills	1.34 ± 0.54	-									
2. AMPS: process skills	0.56 ± 0.64	0.827*** (0.777***)	-								
3. Visual-motor integration	93.98 ± 25.34	0.546*** (0.526***)	0.563*** (0.512***)	-							
4. Visual discrimination	8.41 ± 3.81	0.309* (0.257)	0.306* (0.235)	0.581***	-						
5. Visual memory	8.77 ± 4.47	0.274* (0.234)	0.277* (0.163)	0.474***	0.361**	-					
6. Visual spatial relationships	9.23 ± 5.10	0.435*** (0.342*)	0.432*** (0.354*)	0.438***	0.410**	0.226	-				
7. Form constancy	7.48 ± 3.82	0.204 (0.224)	0.162 (0.104)	0.191	0.171	0.244*	0.275*	-			
8. Visual sequential memory	7.88 ± 4.94	0.482*** (0.368**)	0.491*** (0.411**)	0.400**	0.432***	0.363**	0.470***	0.499***	-		
9. Figure ground	9.33 ± 3.96	0.160 (0.135)	0.138 (0.044)	0.262*	0.162	0.201	0.263*	0.300*	0.238	-	
10. Visual closure	7.71 ± 3.81	0.171 (0.117)	0.177 (0.160)	0.320**	0.290*	0.501***	0.147	0.145	0.122	0.148	-

AMPS = Assessment of motor and process skills. Partial correlation controlling for IQ and age (in parentheses).

* *p* < 0.05;

** *p* < 0.01;

*** *p* < 0.001.

To examine the extent to which motor-free visual perception abilities predict self-care performance, an analysis was conducted by using a stepwise multiple regression analysis with AMPS motor skills and AMPS process skills as dependent variables and four motor-free visual perception subtests as independent variables. In step 1, the sequential memory scores of young children with ASD were selected in the equation, with adjusted R^2^ = 0.220, *F* (1, 64) =19.37 and *p* < 0.001 (Table 2). Sequential memory and visual spatial relationships scores were selected in step 2. Visual discrimination and visual memory were not shown to account for a significant portion of the variance in self-care and were excluded from further analysis. Overall, sequential memory and visual spatial relationships accounted for 26.5% of the variance in AMPS motor skills. For AMPS process skills, the sequential memory scores of young children with ASD were selected in the equation, with adjusted R^2^ = 0.229, F (1, 64) =20.29 and *p* < 0.001 in step 1. The sequential memory and visual spatial relationships were selected in step 2 and accounted for 27.0% of the variance.

**Table 2:** Stepwise multiple regressions of motor-free visual perception on self-care performance.

Variables	B	*β*	*p*	R^2^	Adjusted R^2^	Change in R^2^	F	*p*
AMPS: motor skills								
Step 1								
Sequential memory	0.052	0.482	<0.001	0.232	0.220	0.232	*F* (1,64) = 19.37	< 0.001
Step 2								
Sequential memory	0.039	0.356	0.004					
Visual spatial relationships	0.028	0.267	0.030	0.288	0.265	0.056	*F* (2,63) = 12.73	0.030
AMPS: process skills								
Step 1								
Sequential memory	0.064	0.491	<0.001	0.241	0.229	0.241	F (1,64) = 20.29	< 0.001
Step 2								
Sequential memory	0.048	0.369	0.003					
Visual spatial relationships	0.032	0.259	0.035	0.293	0.270	0.052	F (2,63) = 13.05	0.035

Unstandardized regression coefficients; *β*: Standardized regression coefficients.

**Figure 1:**
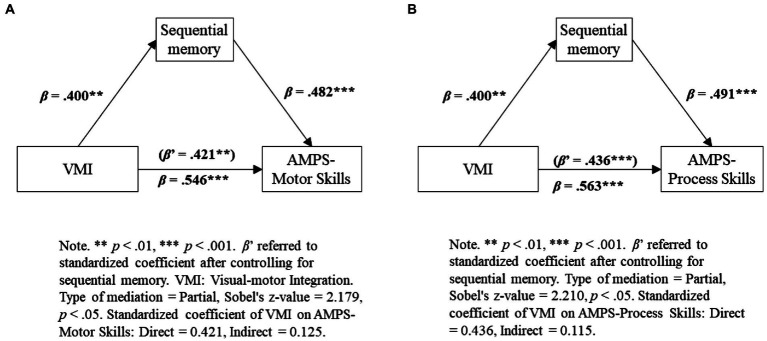
**(A)** and **(B)** Results of testing for mediation by sequential memory on relationship between visual-motor integration and self-care performance.

To examine whether sequential memory and visual spatial relationships were potential mediators of the relationship between visual-motor integration and self-care performance, we followed the method outlined by Hayes (2012). A series of regression analyses were performed to test for mediation. Table 3 confirms that sequential memory mediated the relationship between visual-motor integration and self-care performance by using Sobel’s test. The mediation effect was significant. Figure 1 presents the mediation models. The greater self-care performance in participants with greater visual-motor integration was accounted for by their greater sequential memory ability (Table 3). No mediation effect was evident for spatial relation ability.

**Table 3:** Regression for mediation analysis.

Mediator	Effect of VMI on mediator (a path)	Unique effect of mediator (b path)	Direct effect (c’)	Indirect effect (ab)	Complete model	Sobel’s test
AMPS: motor skills (c path) *β* = 0.546, SE = 0.002, *p* < 0.001						
Sequential memory	0.400 (0.022)** [0.033, 0.123]	0.482 (0.012)*** [0.029, 0.076]	0.421 (0.002)** [0.011, 0.058]	0.125** [0.001, 0.005]	R^2^ = 0.381, F(2, 63) = 19.403***	z = 2.179*
Visual spatial relationships	0.438 (0.023)*** [0.043, 0.134]	0.435 (0.012)*** [0.022, 0.069]	0.441 (0.002)*** [0.005, 0.014]	0.105** [0.0004, 0.005]	R^2^ = 0.346, *F*(2, 63) = 16.640***	z = 1.824
AMPS: process skills (c path) *β* = 0.563, SE = 0.003, *p* < 0.001						
Sequential memory	0.400 (0.022)** [0.033, 0.123]	0.491 (0.014)*** [0.033, 0.123]	0.436 (0.003)*** [0.013, 0.069]	0.115** [0.001, 0.006]	R^2^ = 0.401, F(2, 63) = 21.046***	z = 2.210*
Visual spatial relationships	0.438 (0.023)*** [0.043, 0.134]	0.432 (0.014)*** [0.026, 0.082]	0.462 (0.003)*** [0.006, 0.017]	0.084* [−0.0001, 0.006]	R^2^ = 0.359, F(2, 63) = 17.663***	z = 1.769

VMI: Visual-motor Integration. All coefficients reported for paths are standardized slopes with the corresponding standard error of the slope in parentheses. 95% CI in square brackets [lower limit of confidence interval, upper limit of confidence interval].

* *p* < 0.05;

** *p* < 0.01;

*** *p* < 0.001.

## Discussion

This study extends the current knowledge of the relationship between motor-free visual perception skills and self-care performance of young children with ASD. There were three main findings. First, self-care performance was significantly correlated with four motor-free visual perception skills: visual discrimination, visual memory, visual spatial relationships, and visual sequential memory skills. Second, sequential memory and visual spatial relationships skills were the main factors related to self-care performance. Third, sequential memory was a mediator of the relationship between visual-motor integration and self-care performance.

According to previous studies, visual discrimination, visual memory, visual sequential memory, figure-ground, visual closure, and visual-motor integration are significantly related to self-care performance (Parush et al., 1998; Boyd and Dawson, 2000; Jasmin et al., 2008; Elbasan et al., 2011; James et al., 2015). Consistent with the previous findings, visual discrimination, visual memory, visual sequential memory, and visual-motor integration were found to relate significantly to self-care performance in young children with ASD. The main differences from previous studies were that figure-ground and visual closure had no significant correlations with self-care, and that visual spatial relationships ability was significantly related to self-care performance in children with ASD. These findings suggest that, when performing self-care tasks, the processing of visual information and the skills and strategies used by young children with ASD are critical. For instance, visual spatial relationships have been shown to be correlated with the performance of self-care in patients with dementia (Marquardt et al., 2011; Bouchard et al., 2013). This is because a better recognition of the spatial and item layout within the environmental surroundings results in better self-care performance.

In a critical review, children with ASD were indicated to have demonstrated superior perception in distance matching and the recall of routes (Smith, 2015), which could make them proficient at recognizing spatial and item layouts in their surroundings. Effective movements and high-quality performance in hand-eye coordination, the critical components for performing self-care tasks well, also rely on strong visual spatial relationships skills (Crawford et al., 2004; Nadkarni et al., 2012; Hwang et al., 2014), which may be why visual spatial relationships have a significant positive correlation with self-care in young children with ASD. As previously mentioned, the effective methods for training self-care skills currently in use are picture schedules, modeling, and observational learning. Imitation places high demands on motor perception and sequential memory (Lopes and Santos-Victor, 2005; Jones and Herbert, 2006; Martin, 2006; Hammill et al., 2014; Vetrayan et al., 2015; Loucks et al., 2016; Brown et al., 2019). Thus, sequential memory and visual spatial relationships are the main factors related to performing self-care tasks.

The best two subtest scores within motor-free visual perception were figure-ground and visual spatial relationships among these young children with ASD. Children with ASD who did effectively use their dominant visual spatial relationships skills may result in better self-care performance. However, better figure-ground skills were not an advantage among these young children with ASD. Notably, sequential memory plays a critical role in performing self-care tasks. Nevertheless, the sequential memory skills were relatively weak in comparison with figure-ground and visual spatial relationships. In this study, we found that visual sequential memory has a mediating effect between visual-motor integration and self-care performance. Visual sequential memory involves looking at, remembering, and recalling a sequence of objects and/or events in the correct order (Martin, 2006). Children with ASD rely more heavily on visual sequential memory skills when performing self-care tasks involving motor integration. According to Gibson’s affordance theory (Gibson, 1979), he stated that “we must perceive in order to move, but we must also move in order to perceive” (p. 223). This fits with the visual perception abilities and visual-motor integration abilities being linked with each other. Some researchers reported that the two skill sets are related and interdependent systems, where visual perceptual abilities are reflected in motor responses (Murphy and Gliner, 1988; Sigmundsson and Hopkins, 2005). It is thus plausible to assume that motor abilities could be a determinant of visual perception abilities. Brown (2012) demonstrated that visual sequential memory and visual-motor integration are related and dependent on one another in children. The results were consistent with the findings of Günal et al. (2019), who also indicated that school-aged children with ASD had substantial fine motor, visual perception, and visual-motor integration difficulties, which could impact their performance of self-care tasks. One of the impediments to self-care performance in children with ASD was mediated through visual sequential memory skills, thus suggesting the need for occupational therapy interventions that target the visual-motor integration and daily living skills of young children with ASD, as well as their visual sequential memory ability.

This study has several limitations worth noting. First, the participants enrolled in this study were mainly mild-to-moderate autism symptoms and did not include the full spectrum. Therefore, caution should be taken when inferring the findings of this research apply to young children with ASD in general. Second, the visual perception assessments adopted in this research, both the TVPS-3 and DTVP-3, were paper-and-pencil tests, which may have difficulties reflecting some unique visual perception characteristics in these young children with ASD, such as superior visual searching, superior route recognition, and inferior motor perception. Future research could design additional performance tests to assess the functional visual perception in young children with ASD. Third, the results of this study represent only the current situation of the children. Early childhood is a dramatic phase in which both development and performance change. Although there were some limitations, the relationships between specific motor-free visual perception abilities and self-care performance in young children with ASD were established in the present research. The results will help researchers learn more about the role of specific motor-free visual perception abilities in young children with ASD.

## Conclusion

This study assessed the relationship between specific motor-free visual perception abilities and self-care performance. This study found that self-care and four motor-free visual perception abilities in young children with ASD were linked. Our current findings demonstrate visual sequential memory mediates the effects of visual-motor integration on self-care performance. To link the findings to the interventions on self-care and visual perception among young children with ASD, further evidence-based research will be warranted.

## Data availability statement

The raw data supporting the conclusions of this article will be made available by the authors, without undue reservation.

## Ethics statement

The studies involving human participants were reviewed and approved by the Institutional Review Board of National Cheng Kung University Hospital. Written informed consent to participate in this study was provided by the participants’ legal guardian/next of kin.

## Author contributions

L-YL conceptualized and designed the study, coordinated and supervised the data collection and coordinated funding acquisition, methodology, project administration, and supervision. I-JC and Y-SS performed the statistical analyses, drafted the initial manuscript, and taken responsibility for the paper as a whole. All authors contributed to the article and approved the submitted version.

## Funding

This study was supported by grants from Ministry of Science and Technology of Taiwan (MOST 109-2221-E-006-142 and 110-2511-H-006-010-MY2).

## Conflict of interest

The authors declare that the research was conducted in the absence of any commercial or financial relationships that could be construed as a potential conflict of interest.

## Publisher’s note

All claims expressed in this article are solely those of the authors and do not necessarily represent those of their affiliated organizations, or those of the publisher, the editors and the reviewers. Any product that may be evaluated in this article, or claim that may be made by its manufacturer, is not guaranteed or endorsed by the publisher.
